# Unraveling the Role of Epithelial–Mesenchymal Transition in Adenoid Cystic Carcinoma of the Salivary Glands: A Comprehensive Review

**DOI:** 10.3390/cancers15112886

**Published:** 2023-05-24

**Authors:** Cosima C. Hoch, Fabian Stögbauer, Barbara Wollenberg

**Affiliations:** 1 Department of Otolaryngology, Head and Neck Surgery, School of Medicine, Technical University of Munich (TUM), 81675 Munich, Germany; cosima.chiara.hoch@tum.de; 2 Institute of Pathology, School of Medicine, Technical University of Munich (TUM), 81675 Munich, Germany; fabian.stoegbauer@tum.de

**Keywords:** epithelial–mesenchymal transition, mesenchymal–epithelial transition, epithelial–mesenchymal plasticity, salivary adenoid cystic carcinoma, metastasis, biomarkers

## Abstract

**Simple Summary:**

Salivary adenoid cystic carcinoma (SACC) is an aggressive tumor that exhibits a neurotropic growth pattern by invading nerve fibers and demonstrates a high tendency for both local spread and early distant metastases. The limited availability of successful chemotherapeutic regimens represents a major obstacle in the efficient treatment of SACC patients. Through epithelial–mesenchymal transition (EMT), cancer cells gain increased mobility and invasiveness, facilitating their ability to infiltrate the basement membrane and disseminate to distant sites in the body. This manuscript aims to review the latest research on potential biomarkers that contribute to the development of EMT, which can aid in identifying new therapeutic targets and improving the oncological management of SACC patients.

**Abstract:**

Salivary adenoid cystic carcinoma (SACC) is considered a challenging malignancy; it is characterized by a slow-growing nature, yet a high risk of recurrence and distant metastasis, presenting significant hurdles in its treatment and management. At present, there are no approved targeted agents available for the management of SACC and systemic chemotherapy protocols that have demonstrated efficacy remain to be elucidated. Epithelial–mesenchymal transition (EMT) is a complex process that is closely associated with tumor progression and metastasis, enabling epithelial cells to acquire mesenchymal properties, including increased mobility and invasiveness. Several molecular signaling pathways have been implicated in the regulation of EMT in SACC, and understanding these mechanisms is crucial to identifying new therapeutic targets and developing more effective treatment approaches. This manuscript aims to provide a comprehensive overview of the latest research on the role of EMT in SACC, including the molecular pathways and biomarkers involved in EMT regulation. By highlighting the most recent findings, this review offers insights into potential new therapeutic strategies that could improve the management of SACC patients, especially those with recurrent or metastatic disease.

## 1. Introduction

Salivary adenoid cystic carcinoma (SACC) is considered the second most frequent malignant salivary gland neoplasm and exhibits a controversial and poorly understood biological behavior, characterized by slow and indolent growth [1,2]. SACC typically arises from the submandibular gland and minor salivary glands, while its occurrence in the parotid gland is relatively rare [3]. SACC has an incidence rate of approximately 4.5 cases per million individuals and constitutes 10% of all salivary gland tumors (SGTs). This type of cancer exhibits three distinct histological growth patterns, namely cribriform, tubular, and solid patterns. Among these patterns, the solid pattern represents the most aggressive form of SACC with an increased risk of metastasis, resulting in shorter disease-specific survival [4].

Despite being uncommon, multiple research studies have investigated the molecular pathogenesis, genetic mutations, and overexpression of proteins in SACC [5,6,7]. The latest advancements in whole genome and exome sequencing have significantly enhanced our understanding of SACC pathogenesis. The MYB-NFIB fusion gene, which results from a balanced translocation, seems to be a crucial feature of SACC. Additionally, sequencing has identified a plethora of driver genes with mutations in downstream pathways that are shared by other extensively researched types of cancer [6].

The regional recurrence rate of SACC has been reported to be 74%; however, relapses appear to occur at a much lower rate compared to distant metastases (DM) [8]. In fact, the 5-year, 10-year, and 15-year overall survival rate in patients with DM are 46%, 19%, and 15%, respectively [9]. The lung is the primary location for DM, with the bone, liver, and brain following closely behind in frequency [10]. Although surgical dissection and postoperative radiotherapy can usually control the local spread of the disease, they are not sufficient to prevent DM, and the outcomes of treatment remain unsatisfactory. Moreover, the most effective systemic chemotherapy protocols remain to be elucidated [11].

Epithelial–mesenchymal transition (EMT) plays a well-documented role in the developmental programs involved in the formation of new tissues and organs, and is typically followed by the reverse event of mesenchymal–epithelial transition (MET) [12,13,14]. The dynamic combination of both processes is subsumed by the term ‘epithelial–mesenchymal plasticity’ (EMP) [15]. In conjunction with the loss of apical–basal polarity and the degradation of epithelial cell–cell contacts, including tight junctions (TJs), adherens junctions (AJs), and desmosomes, the architecture of the actin cytoskeleton in cells undergoing EMT is remodeled to adopt a spindle-shaped mesenchymal morphology and acquire the ability to migrate and invade through the formation of protrusions [12]. Thus, while EMT is crucial for invasion, dissemination, and extravasation, the reverse process of MET engages in metastatic colonization [16]. Multiple oncogenic signaling pathways and the induction of hypoxia regulate EMT through the activation of transcription factors (EMT-TFs), such as SNAIL (also SNAI1) and SLUG (also SNAI2), the basic helix–loop–helix factors TWIST1 and TWIST2, the zinc finger E-box binding homeobox factors ZEB1 and ZEB2, and other EMT-TF-associated molecules [17].

Owing to the limited mechanistic understanding of the SACC pathogenesis and the scarcity of effective chemotherapeutic regimens, surgery and/or radiation therapy remain the primary treatment modalities for these patients. As a result, treatment of SACC is generally associated with significant morbidity and debilitating facial deformity. Deciphering the molecular principles driving EMT may help in the identification of new avenues for an improved understanding of tumor cell plasticity. Its response to novel and established therapeutic modalities has the potential to reveal new targets for the more effective, less toxic, and personalized treatment of SACC patients. In this review, we discuss the potential biomarkers underlying the development of EMT and summarize the most recent evidence on this complex research topic for SACC.

## 2. Multiple Molecular Signals Driving EMT in Cancer Progression and Metastasis

Metastasis, the spread of cancer from the primary site to distant locations in the body, involves a complex series of events. These stages include invasion, intravasation, extravasation, and metastatic colonization. The initial step of invasion and migration determines the rate at which the metastasis process occurs [18]. Cancer cells exploit the process of EMT to obtain an invasive phenotype and detach from the primary tumor [19]. During EMT, there is a decrease in cell adhesion molecules such as E-cadherin (coded by CDH1) and cytokeratin, while mesenchymal markers such as vimentin (coded by VIM), N-cadherin (coded by CDH2) and fibronectin (coded by FN1) increase [20]. EMT is activated by pleiotropic intrinsic and extrinsic factors and is governed temporally and spatially, with EMT-TFs such as SNAIL/SLUG, TWIST1/TWIST2, and ZEB1/ZEB2 playing a fundamental role. These factors can inhibit the expression of epithelial genes, including the CDH1 gene, by attaching to E-box motifs in corresponding promoter regions [12,21]. Concurrently, EMT-TFs trigger genes linked to mesenchymal characteristics such as VIM, FN1, and CDH2, either directly or indirectly [14]. MicroRNAs (miRs), which are noncoding RNAs, have the ability to selectively bind mRNA and either promote its degradation or inhibit its translation. Due to their ability to directly regulate the SNAIL family, a variety of miRs can impact the progression and metastasis of EMT. Thus, alterations in miR expression can have a significant effect on EMT regulation [22]. Multiple signaling pathways, such as transforming growth factor ß (TGF-ß), Wnt, Notch, and PI3K-AKT, participate in the regulatory network of EMT. Additionally, post-translational modifications can trigger EMT and facilitate tumor cell metastasis. Epigenetic modifications operate in controlling the expression of EMT-TFs, which in turn regulate metabolic pathways, transcription, differentiation, and apoptosis during the EMT process [12,23].

Given its high level of plasticity, EMT not only facilitates the formation of macrometastasis during malignant progression, but also allows cells to exhibit various EMT modes, depending on the microenvironment and the prevailing state of the tissue, resulting in very distinct phenotypes [24]. Notably, carcinoma cells rarely lose all epithelial characteristics and acquire a full spectrum of mesenchymal features. Rather, this refers to what is known as partial EMT: an incomplete transition to a mesenchymal cell that is encountered in developmental settings and often leads to the maintenance of partial cell–cell junctions [25]. This concurrent acquisition of migratory capacity alongside the maintenance of intercellular adhesion is linked to the collective migration of many cell populations [25]. Conversely, carcinosarcomas are an important, albeit rare exception in which distinct epithelial and mesenchymal compartments coexist and derive from a common cellular progenitor [26].

Although EMT might facilitate the spread of cancer cells from primary tumors, a growing body of research indicates that MET and mesenchymal cell re-epithelialization fulfills an important function in the growth of secondary tumors. While the mechanisms underlying MET appear to be far less well understood than the underlying mechanisms that drive EMT, it remains to be elucidated how the mechanisms driving these processes are interrelated. Instead of stimulating MET by activating a specific transcription factor or signaling pathway, in many in vitro and in vivo cancer studies, MET is activated by silencing an EMT-inducing signal such as TGF-ß or by downregulating an EMT-TF [27,28,29,30]. Epithelial differentiation during carcinogenesis can also be achieved by inducing the c-met proto-oncogene (c-MET) [31]. Additionally, other factors, including Frizzled-7, a receptor for the canonical Wnt signaling pathway, 5-azacytidine, a DNA methyltransferase inhibitor, and the stable RNA interference-mediated inhibition of SNAIL expression, have been shown to drive a complete MET program in carcinoma cell lines [32,33,34].

## 3. The Role of EMT-TFs in Cancer Invasion and Metastasis in SACC

EMT-TFs serve the purpose of maintaining stem cell characteristics, increasing the tumorigenicity of cells, and connecting to cancer stem cells. Furthermore, EMT-TFs create a phenotype that fosters cell survival by supporting DNA repair, evading immune recognition, resisting treatments, delaying aging, and avoiding cell death, which is advantageous under various stressful conditions. Taken together, the classical EMT functions and the varied, context-dependent, unique, and non-classical functions of EMT-TFs, which are also dynamically modulated by the tumor microenvironment, allow cancer cells to continuously adjust to changing conditions [35]. Table 1 provides an overview of different EMT-TF expressions, their original function, and participation in EMT in SACC.

### 3.1. TWIST Expression in SACC and Its Significance in Pathogenesis and Prognosis

TWIST is a crucial protein that drives the EMT process by decreasing the expression of E-cadherin and increasing the expression of mesenchymal markers [13]. TWIST consists of two proteins, namely TWIST1 and TWIST2, that exhibit comparable levels of expression in tumors [36]. Studies revealed TWIST to be the most highly expressed factor among EMT-TFs in SACC tumor tissue samples, with the solid variant being the most reactive [37,39]. These results agree with a study performed by Shen et al., which showed significantly higher TWIST expression in SACC compared to pleomorphic adenomas (PAs) and normal parotid gland tissues [38]. Additionally, TWIST expression was documented to be higher in cases with DM than in those without DM [38].

The overexpression of TWIST has been observed to prompt an EMT-like transformation of metastatic cell lines of human SACC, leading to upgraded migration and invasion capabilities [39]. Elevated TWIST expression has also been strongly tied to perineural invasion (PNI), local regional recurrence, and DM of SACC. A high, predominantly nuclear TWIST expression in SACC was recorded when compared to mucoepidermoid carcinoma (MEC), normal glands, and benign tumors [40]. Kerche et al. similarly detected broad nuclear expression of the TWIST protein in SACC, although they did not find any link between TWIST expression and the absence of E-cadherin expression [41]. These findings corroborate previous research conducted on various types of tumors, which demonstrated a possible association between the elevated nuclear expression of TWIST and the increased invasiveness of tumors [42,43].

Recently, siRNA was used to reduce TWIST levels in SACC cells in order to investigate how TWIST is involved in the neurophilic invasion of these cells. Inhibiting TWIST resulted in decreased cancer cell migration, invasion, and PNI ability, as well as a reduction in the expression of genes associated with EMT and Schwann cell markers. When S100A4 was overexpressed using a plasmid, researchers noted a significant growth in the migratory, invasive, and PNI ability of SACC cells, along with a more fibroblast-like appearance and an increase in pseudopodia formation [44].

### 3.2. SNAIL and SLUG: Key Transcription Factors Driving EMT and Metastasis in SACC

According to broad evidence, the SNAIL transcription factor suppresses the expression of E-cadherin, which results in the inhibition of cell–cell adhesion and augmented cancer invasion in different types of cancer [46,47]. SNAIL has a significant role in triggering the process of EMT and its reactivity was shown to be heterogeneous, with a more distinct nuclear pattern in the solid variant and invasive tumor front of SACC [37,48]. Remarkably, high levels of SNAIL expression are linked to PNI, local and regional recurrence, as well as DM of SACC [48]. Zhao et al. pointed out that the increased expression of SNAIL and Integrin-linked kinase (ILK) strongly correlated with the solid pattern, advanced TNM stages, a high risk of recurrences, and DM [52]. Of note, the authors found a correlation between the overexpression of SNAIL and N-cadherin and a neural invasive phenotype of SACC.

Another protein, the small heat-shock protein of 27 kDa (HSP27), has also been connected to cancer progression in SACC. The overexpression of HSP27 in SACC cells encouraged migration and invasion, induced EMT, and upregulated the expression of SNAIL and paired related homeobox protein 1 (PRRX1) [53]. In contrast, HSP27 silencing reduced migration and invasion and contributed to the MET of SACC cells. Additionally, HSP27 downregulated the expression of E-cadherin indirectly via the activation of SNAIL and PRRX1 expressions, while the overexpression of SNAIL or PRRX1 restored migration and invasion in HSP27 knockdown cells. The results of the study align with the clinical outcome of patients with SACC, as HSP27 expression is related to radioresistance and the poor prognosis of SACC patients, as well as with the expression of PRRX1 or SNAIL in SACC tissues.

More recently, Du et al. identified protein kinase D1 (PKD1) as a key regulator of SACC progression [55]. The inhibition of PKD1 resulted in the decreased proliferation, migration, invasion, and EMT of SACC cells. Conversely, the overexpression of kinase-active PKD1 evoked EMT and promoted cell migration in human SACC cells. The downregulation of PKD1 was found to control SNAIL via phosphorylation at Ser-11 on SNAIL protein and the promotion of proteasome-mediated degradation, which ultimately reduced lung metastasis in vivo.

Belulescu et al. reported that SLUG was the second most abundant EMT-TF detected in SACC, with reactivity observed in 78% of cases, mainly in the stroma at the level of cancer-associated fibroblasts (CAFs) and endothelial cells [37]. SLUG expression was noted to be higher at the invasive front and in cases associated with DM and lymph node metastases [37]. Similarly, Tang et al. stated that 72% of studied SACC exhibited reactivity to SLUG, and this correlated significantly with TNM stages, PNI, local regional recurrence, and DM [56]. The mitogen-activated protein kinase (MAPK)–SLUG pathway has been shown to contribute to the metastatic formation of SACC [58]. The high expression of SLUG and the extracellular matrix metalloproteinase inducer (EMMPRIN), along with low E-cadherin expression, were significantly linked with PNI and SACC [59]. Silencing SLUG inhibited the EMT process by reducing EMMPRIN expression and subsequently increasing E-cadherin expression in SACC. Two studies also revealed that elevated levels of BTBD7, a spliced variant of BTBD containing binding sites for significant transcription factors, were significantly linked to SLUG expression in SACC [60,61]. The reduction in BTBD7 considerably impeded the expression of SLUG in SACC cells. Furthermore, Yang et al. found a significant difference in SLUG expression between SACC and normal salivary gland cases, which was significantly linked with TNM stage, tissue typing, and DM.

Liu et al. displayed evidence that the epiregulin (EREG)–epidermal growth factor receptor (EGFR)–SNAIL/SLUG pathway propels EMT and metastatic characteristics in SACC cells, suggesting it could be an effective target for treating metastatic SACC [62]. In accordance with these data, epidermal growth factor (EGF) stimulation resulted in EGRF activation, which stabilized SNAIL expression and provoked EMT [63]. The suppression of SNAIL expression significantly inhibited EGF-induced EMT in these tumors. Jiang et al. further supported the aforementioned findings, as the authors displayed that the EMT process in SACC cell lines was reversed by pretreatment with Nimotuzumab, an anti-EGFR monoclonal antibody [64]. Moreover, high levels of BMI-1, nuclear/cytoplasmic SNAIL and SLUG expression, and low membranous E-cadherin expression were significantly coupled with DM in SACC [65].

### 3.3. ZEB1/ZEB2: A Key Regulator of EMT in SACC

ZEB1/ZEB2 is a critical regulator in various tumor types and is downstream of nuclear factor kappa B (NF-κB) [66,67]. However, the immunohistochemical (IHC) detection of ZEB1 in SACC tumor parenchyma did not depict any positive reaction, while positive staining was examined in the tumor stroma at the level of CAFs and endothelial blood cells in all cases [37]. These results align with a study carried out by Kerche et al., that observed ZEB1 expression on both tumor cells and stromal cells [41]. Moreover, NF-κB directly binds to the promoter region of ZEB1, leading to its transcriptional activation [68]. Insulin-like growth factor-binding protein 2, which is significantly upregulated in SACC, plays a crucial role in the invasion and metastasis of this cancer type by modulating the NF-κB/ZEB1 signaling pathway. Interestingly, the knockdown of cancer cell-derived immunoglobulin G (cancer-IgG) in SACC suppressed EMT by upregulating E-cadherin and downregulating ZEB1/ZEB2, suggesting that cancer-IgG could be a useful prognostic marker for this disease [69]. Additionally, cancer-IgG overexpression was significantly correlated with metastasis, recurrence, and invasion in SACC.

## 4. Expression of Major EMT Markers in SACC

**Figure 1:**
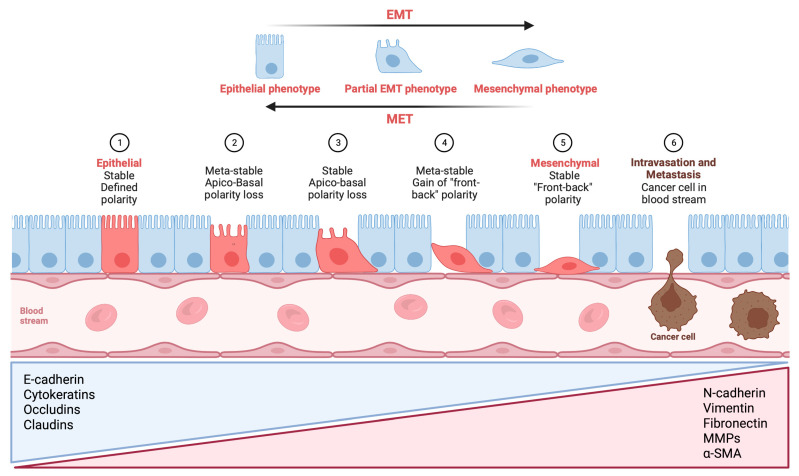
Visual illustration of a typical epithelial–mesenchymal transition (EMT) program. Epithelial cells exhibit apical–basal polarity and express specific molecules associated with the maintenance of cell polarity and the epithelial state, which are listed in the blue triangular box. As epithelial cells undergo a progressive loss of their distinctive characteristics, they acquire mesenchymal features while retaining certain epithelial traits. In certain situations, they may acquire a complete set of mesenchymal features. Mesenchymal cells display front-to-back polarity, with a highly reorganized cytoskeleton, and they express a distinct set of molecules that promote and preserve the mesenchymal state (listed in the red triangular box). As a result, cells undergoing EMT exhibit increased migration, invasion, and stem-like properties, which can result in treatment resistance, metastasis formation, and recurrences in patients, ultimately affecting their clinical outcomes. Metastatic colonization requires a reversal of EMT, referred to as mesenchymal-to-epithelial transition (MET), which facilitates the expansion of tumor cells, an essential prerequisite for metastatic growth. E-cadherin: epithelial cadherin; N-cadherin: neural cadherin; MMP: matrix metalloproteinase; α-SMA: alpha-smooth muscle actin (Figure created using BioRender, Toronto, ON, Canada).

Epithelial cells are transdifferentiated towards a mesenchymal cell phenotype during EMT, as they lose epithelial markers such as E-cadherin or various types of cytokeratin (CK), while gaining mesenchymal markers such as vimentin, fibronectin or N-cadherin [70]. Figure 1 provides a visual illustration that outlines the various phases of EMT.

### 4.1. Expression of CK in SACC

CK is a group of intermediate filaments that offer structural reinforcement to epithelial cells. CK expression is dependent on the tissue or organ and the degree of cell differentiation. Due to the specific cytokeratin patterns associated with different cellular sources, CK can be employed as a diagnostic marker for tumors, particularly epithelial cancers [71,72].

To characterize the CK profile of SACC, several studies have employed IHC examination of CK reactivity. In one study of 32 SACC cases, AE1/AE3 staining was found to be most intense in solid histological subtypes, followed by the tubular and cribriform subtypes [73]. SACC cells demonstrated strong and widespread positivity for AE1/AE3, comparable to the residual parenchyma of the major or minor salivary gland [74]. Furthermore, SACC exhibited positivity for CK7 but negativity for CK20, consistent with the results reported by Lee et al. [75]. Ben Salha et al. observed that AE1/AE3 and CK7 showed widespread and positive reactivity in solid SACC, whereas CK5/6 and CAM5.2 were only reactive in some neoplastic cells [76]. Notably, CK14-positive cells at the invasive front of SACC were reported to stimulate cancer cell invasion and may serve as a potential prognostic marker in these patients [77].

### 4.2. The Role of E-Cadherin in SACC: Implications for Tumor Invasion, Metastasis, and EMT

E-cadherin is a critical protein that plays a vital role in maintaining the structural integrity of epithelial tissue and is involved in regulating epithelial cell growth, differentiation, and survival. Multiple research projects have proven a reduction in E-cadherin expression in specimens of SACC compared to adjacent normal salivary parenchyma, leading to PNI, regional recurrence, and DM [41,44,52,78,79,80,81]. These studies revealed that regardless of the histological subtype, SACC cells located at the invasive tumor front and near nerve fibers exhibit reduced E-cadherin expression, with the alteration of the subcellular pattern from membranous to predominantly cytoplasmic [73,82]. Interestingly, the co-expression of E-cadherin with the gene cadherin 4 (CDH4), encoding retinal cadherin (R-cadherin), inhibits the growth and metastasis of SACC [83]. Moreover, the overexpression of ILK via the dysregulation of E-cadherin/N-cadherin might trigger EMT and thus facilitate SACC invasion and metastasis. Notably, a reduction in E-cadherin expression has also been explored in the myoepithelial cells of SACC compared to normal gland tissue [41,84], and the absence of E-cadherin expression correlated with PNI, vascular invasion, and DM in SACC patients.

In contrast to previous studies, two groups of researchers have observed E-cadherin expression in most of their SACC samples, suggesting that EMT in SACC could be related to the transformation of ductal epithelial cells into myoepithelial cells [85,86]. However, van der Wal et al. reported a favorable prognosis of SACC patients with diminished E-cadherin expression [87]. Wu et al. conducted a study on the invasion of neoplastic epithelial cells into adjacent tissue in SACC samples and noticed that the cells invaded as multicellular units and maintained their expression of E-cadherin [88]. The study’s IHC results also showed that cancer cells at the invasive front did not exhibit a complete EMT signature. The three-dimensional (3D) spheroid invasion assay revealed that leader cells displayed an elongated phenotype while still retaining E-cadherin expression, representing a partial EMT phenotype. These findings are consistent with previous studies that have noted that leader cells lack a complete EMT signature and cannot be identified through canonical mesenchymal markers [89,90]. EMT is not usually uniformly evident throughout the entire tumor tissue, and partial EMT is more common in cancer than complete EMT [91,92].

### 4.3. Vimentin: A Key Intermediate Filament in Mesenchymal Cells and Its Role in SACC Metastasis

Vimentin is a type of intermediate filament that is exclusively present in mesenchymal cells. It is a key structural protein that is widely distributed in different types of cells, including fibroblasts, vascular endothelial cells, smooth muscle cells, cartilage and bone cells, and nerve sheath cells, that form the connective tissue [71,93].

Belulescu et al. reported that the solid variant of SACC exhibited a more prominent EMT process, likely due to the more obvious vimentin expression perceived in these cases [73]. Furthermore, mesenchymal features were more pronounced in neoplastic cells located at the invasive tumor front and those associated with PNI, as demonstrated by Zhang et al., who found enlarged vimentin expression in areas of nerve invasion [44]. In accordance with these results, various authors declared positive vimentin staining in SACC together with the co-expression of keratin in these cells [94,95].

### 4.4. The Complex Role of Fibronectin in SACC: Promoting PNI, Inhibiting Invasion, and Potential Prognostic Marker

Fibronectin is a type of glycoprotein that creates an extracellular matrix. It is a polypeptide that combines to form a dimer with a molecular weight of approximately 250 kDa. Its primary function is to facilitate the attachment of various cell types such as fibroblasts, hepatocytes, and nerve cells. The cell surface-specific receptor known as Integrin plays a significant role in processes such as cell adhesion, migration, and phagocytosis, particularly in the context of tissue damage [71,96].

Similar to the results regarding vimentin expression, fibronectin seems to act as a key player in the process of SACC invasiveness. In solid subtypes, fibronectin expression was observed to diminish in the outer region of invasive tumor clusters, while strong expression was detected in neoplastic cells that surround and infiltrate the nerve fibers, suggesting its potential dual role in promoting PNI, while potentially inhibiting invasion at the invasive tumor front [73,97,98,99]. In contrast, other studies have found lower fibronectin expression in the solid SACC variant compared to the tubular and cribriform subtypes [100,101]. Interestingly, the authors found a significant association between fibronectin expression, low TNM stages, and non-metastatic SACC cases [100]. Moreover, they postulated a potential inverse correlation between BTBD7 and fibronectin, indicating BTBD7′s potential prognostic role in predicting the invasion and metastasis of SACC.

### 4.5. N-Cadherin Expression in SACC: Association with PNI and EMT

N-cadherin is a transmembrane glycoprotein that belongs to the classic cadherin superfamily and has a molecular weight of 130-kDa. Its expression has been detected in a range of cell types, such as neurons, endothelial cells, and cardiomyocytes [71,102].

Zhao et al. found that N-cadherin was expressed in 68.1% of the investigated SACC cases, with a membranous and/or cytoplasmic reaction pattern, and the highest expression levels were found in cases with PNI [52]. These findings are supported by Zhang et al., who also reported heightened N-cadherin expression in the area of nerve invasion in SACC [44]. In contrast, Belulescu et al. observed N-cadherin reactivity in only 35% of SACC cases, with low intensity and no significant difference between the three histological subtypes [73]. The discrepancy in results may be due to differences in the sample size, technique, or antibodies used for N-cadherin detection. Additionally, N-cadherin expression was found to be negatively aligned with E-cadherin expression and positively associated with the expression of SNAIL and ILK in SACC, implying that ILK overexpression might induce EMT by dysregulating the expression of these cadherins, leading to SACC invasion and metastasis [52]. Jiang et al. reported that thioredoxin 1 (TXN) and thioredoxin reductase 1 were overexpressed in SACC cases with metastasis and were partnered with high N-cadherin expression and low E-cadherin expression, proposing that TXN incites an EMT-like phenotype that promotes SACC migration and invasion [103]. These findings highlight the potential of N-cadherin, SNAIL, ILK, and TXN as prognostic markers for SACC.

## 5. TGF-ß-Mediated EMT in SACC

**Figure 2:**
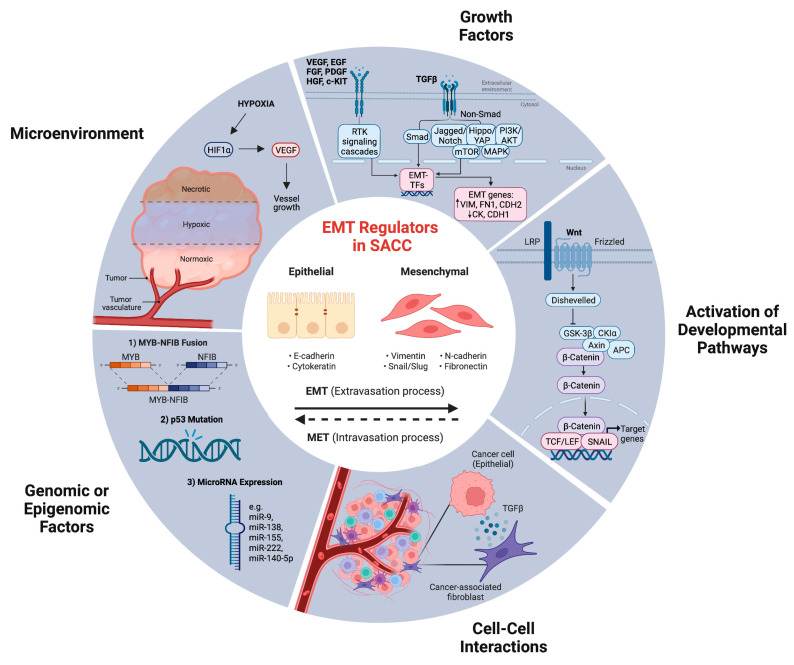
Schematic representation of various mechanisms that have been reported to regulate epithelial–mesenchymal transition (EMT) in SACC patients. Five distinct regulatory mechanisms have been identified, including hypoxia, the activation of growth factors and developmental pathways (such as the transforming growth factor ß (TGF-ß) and Wnt signaling pathway), interaction between cancer-associated fibroblasts and adjacent tumor cells, and genomic or epigenomic alterations (such as MYB-NFIB fusion, p53 mutation, and microRNA (miR) expression). These factors play a significant role in the modulation of the epithelial–mesenchymal spectrum. VEGF: vascular endothelial growth factor; EGF: epidermal growth factor; FGF: fibroblast growth factor; PDGF: platelet-derived growth factor; HGF: hepatocyte growth factor; RTK: receptor tyrosine kinase; mTOR: mammalian target of rapamycin; GSK3β: glycogen synthase kinase-3 beta; CK1a: casein kinase 1a; APC: adenomatous polyposis coli; LEF: lymphoid enhancer-binding factor; TCF: T cell factors; HIF1α: hypoxia-inducible factor 1-alpha. (Figure created using BioRender, Toronto, ON, Canada).

EMT is a complex process that is triggered by distinct signaling pathways in response to external stimuli, with TGF-β being a key player in this process [104] (Figure 2). In particular, the potential involvement of TGF-β in the migration and invasion of in vitro SACC cells via the Smad pathway has been documented by Dong et al. [105]. The same group of authors found that upregulation of TGF-β, phosphorylation of Smad2, and decreased expression of membrane β-catenin were significantly linked to lung metastasis in SACC [106]. Moreover, they proposed that TGF-β-induced EMT might be involved in the mechanisms of SACC metastasis in vivo.

The physical structure of cell–cell attachment is provided by the E-cadherin/β-catenin complex, which is widely recognized [107]. This complex is crucial, as highlighted by Birchmeier et al., who found that if any of the components of the complex were downregulated, the tumor-suppressive actions of AJs would be lost, allowing cancer cells to escape from the primary tumor and migrate to other sites [108]. The loss of the E-cadherin/β-catenin complex was further reported to lead to tumor progression in salivary glands [109]. The cytoplasmic form of β-Catenin influences adhesion, while the nuclear form of β-Catenin acts as a transcription factor and impacts signaling pathways (such as Wnt-signaling) that stimulate the expression of SNAIL, SLUG, and ZEB1 transcription factors, resulting in higher cell mobility and invasion [110].

Ma et al. investigated the relationship between anterior gradient 2 (AGR2), which has been recently linked with hormone-dependent cancers, and TGF-β/CD147 in SACC, and found AGR2 to be positively correlated with TGF-β and CD147, displaying a potential role of AGR2 in EMT [111]. Interestingly, silencing AGR2 in SACC cells exposed to exogenous TGF-β has led to the reduced expression of N-cadherin, SLUG, SNAIL, and CD147, while upregulating E-cadherin.

Jiang et al. conducted a study that demonstrated the involvement of TXN in TGF-β-induced EMT and the promotion of metastasis in SACC. Their findings revealed that TXN stabilizes the transcriptional factors SNAIL and SLUG, and collaborates with the PI3K/Akt/GSK-3β signaling pathway to promote EMT and metastasis in SACC cells [103]. Moreover, reducing the expression of TXN and subsequent treatment with TGF-β resulted in a diminution in the effects of TGF-β on EMT, suggesting that TXN might have a crucial role in the TGF-β-induced EMT in SACC.

## 6. MYB Promotes SACC Metastasis by Regulating EMT

The MYB oncogene has been closely linked to SACC since the discovery of t(6;9) translocations that repeatedly join the MYB and NFIB genes in many of these tumors [112,113]. MYB encodes a transcription factor that binds to DNA and is involved in various types of human malignancies in hematopoietic, epithelial, and neural tissues [114,115,116]. The recurrent t(6;9) translocation leads to the fusion of the MYB gene on chromosome 6 with the NFIB gene on chromosome 9, resulting in either the overexpression of an activated Myb protein or a novel Myb–NFIB fusion oncoprotein.

MYB has been found to be strongly associated with lung metastasis and correlated with the pathological type of tumors in patients diagnosed with SACC [113]. In SACC tissues, MYB expression was revealed to be significantly higher (at a rate of 90%) compared to normal salivary gland tissues [117]. Although this finding is in line with prior research, a more recent study demonstrated that the MYB proto-oncogene to NFIB was negatively connected with CDH1 and positively correlated with VIM [117]. In addition, the authors showed that MYB upregulated EMT-associated markers, such as vimentin, N-cadherin, and α-SMA, in SACC cells, which is consistent with previous studies on breast cancer [118,119].

## 7. Hypoxia as an Essential Factor Related to EMT in SACC

There is mounting evidence suggesting that changes in oxygen levels in the tumor microenvironment and the activation of hypoxic signaling pathways through hypoxia-inducible factors (HIFs) play a crucial role in triggering and regulating EMT (Figure 2). As a result, EMT is now considered a pivotal point where hypoxia and cancer converge [120,121,122]. HIF is a member of the basic helix–loop–helix protein family, consisting of three types: HIF1-α, HIF2-α, and HIF3-α, along with the Hypoxia-Inducible Aryl Hydrocarbon Receptor Nuclear Translocator [123]. Studies in patients with head and neck squamous cell carcinoma have shown that the simultaneous expression of HIF-1α, TWIST2, and SNAIL is related to the highest percentage of metastasis and the poorest prognosis [120].

As SACC research in the field of hypoxia is limited, Wang et al. utilized a three-dimensional culture system to investigate the role of hypoxia in promoting the migration, invasion, EMT, and vascular mimicry (VM) formation of SACC cell lines [124]. The results of the study insinuate that hypoxia plays a central role in these processes. The authors also observed that the overexpression of vascular endothelial growth factor enhanced VM formation, as well as the expression of VE-cadherin, N-cadherin, CD44, and ALDH1 in SACC cells. Another study found that a rise in HIF-2α expression was associated with invasion and metastasis in SACC, and the positive coexpression of HIF-2α/TWIST2 was a significant and independent predictor of a worse prognosis in SACC, as confirmed via multivariate analysis [39].

## 8. The Role of MicroRNAs in the Regulation of EMT Progression in SACC

MiRs are a type of non-coding RNA that are approximately 22 nucleotides long [125]. Numerous investigations have shown that miRs have the ability to manipulate the expression of key proteins that, in turn, modulate post-translational EMT progression, ultimately affecting the regulation of stem cell pluripotency and tumor progression [126,127].

Recent research has highlighted the importance of the altered expression of EMT factors in regulating miRs in SACC, particularly miR-9, miR-138, miR-155, miR-222, and miR-140-5p [41,58,128,129,130,131,132]. A study by Qiao et al. revealed that the overexpression of miR-140-5p reduced the proliferation and invasion of SACC cells, caused apoptosis, and inhibited the growth of SACC tumors in vivo [133]. Conversely, loss-of-function experiments showed that knocking down miR-140-5p increased SACC cell proliferation and invasion, inhibited apoptosis, and accelerated tumor growth in vivo. The researchers also evaluated various mediators involved in EMT, such as E-cadherin, N-cadherin, vimentin, and MMP-2/-9, and found that the overexpression of miR-140-5p suppressed EMT and reduced the protein levels of MMP2 and MMP9. These findings point out that miR-140-5p may have anti-tumor effects on SACC by controlling apoptosis- and EMT-related factors, as well as MMPs. However, to date, only one study has established a correlation between the expression of miRs and EMT markers in SACC [133].

## 9. The Role of c-Kit in EMT Progression of SACC

c-Kit is a receptor that functions as a tyrosine kinase and is activated by its ligand, stem cell factor. It plays a crucial role in regulating developmental processes and can stimulate signaling pathways that are related to preserving progenitor cells [134]. The significance of c-kit signaling in the development of various types of tumors, such as hematopoietic cell tumors, small cell lung cancer, melanoma, gastrointestinal stromal tumor, and colorectal cancer, is well established and is reliant on its activation [135].

In SACC, c-kit signaling has been partnered with the expression of EMT factors such as SLUG, SNAIL, ZEB1, ZEB2, PRRX1, and homeobox B7, which are involved in the progression of the disease [136]. Moreover, TGF-β, a regulator of EMT, can stimulate c-Kit expression in SACC. The level of c-Kit expression has been noted to be greater in the advanced stages of SACC compared to the early stages [56]. In accordance with these findings, Kerche et al. demonstrated c-Kit expression in the ductal areas of SACC [41]. In contrast, a study by Salanihejad et al. did not reveal significant variations in c-Kit expression between benign and malignant SGTs [137].

## 10. p53 as a Potential Therapeutic Target for Inhibiting PNI in SACC

The tumor suppressor gene p53 plays a critical role in inhibiting tumor growth, and its mutations are documented to contribute to the development and progression of various cancers [138,139]. Perineural invasion, an exceptional pathological entity that significantly differs from lymphatic and vascular invasion, is deemed a crucial characteristic of SACC [140]. Recent research by Yang et al. suggests that p53 may carry out a regulatory role in the metastatic process of SACC [141]. The inhibition of p53 expression in SACC cells in vitro led to PNI activity by promoting EMT, including the decreased expression of E-cadherin, EMA, and CK5, and the increased expression levels of vimentin, N-cadherin, and C-cadherin. In this regard, it might also be interesting to investigate whether the targeted augmentation of p53 expression in SACC could reduce EMT and associated PNI.

## 11. Clinical Trials Evaluating Therapies Influencing the EMT-Axis in SACC

Therapies designed to target specific pathways or processes, otherwise known as targeted therapies, have emerged as an auspicious strategy for the treatment of advanced and recurrent neoplasms. Used in conjunction with pharmacological treatment, these targeted therapies can concentrate their effects on the tumor cells, thereby mitigating systemic toxicity. An array of pharmacological agents, including Imatinib (a c-KIT inhibitor), Gefitinib and Cetuximab (EGFR inhibitors), Lapatinib (which inhibits both EGFR-1 and EGFR-2), and Lenvatinib (a multi-targeted kinase inhibitor), which impart therapeutic effects on the EMT axis, have been investigated in the context of SACC treatment [142,143,144,145,146]. Details regarding completed and ongoing phase I/II clinical trials investigating therapies influencing the EMT axis in SACC are presented in Table 2.

In relation to oncological outcomes, a study involving 16 patients with inoperable or metastatic SACC that were evaluated for their response to Imatinib treatment yielded limited positive results [145]. Of the 15 assessable patients, none demonstrated any objective response to the therapy. However, it should be highlighted that, of these 15 patients, 9 exhibited stable disease (SD) as their most favorable response to the therapy. Six patients presented with disease progression (DP) subsequent to two cycles of imatinib administration. In a separate study involving 28 patients diagnosed with recurrent and/or metastatic SACC, the objective response rate to Lenvatinib treatment was assessed [146]. Among the 26 assessable patients, 3 achieved partial responses. Furthermore, 4 of the 20 patients demonstrating SD displayed reductions in their target lesions, ranging from 23% to 28%. With a median follow-up period of 29 months, 2 patients remained on therapy, 10 patients exhibited DP but survived, and 14 patients had passed away due to DP. The median overall survival, progression-free survival, and duration of response were 27 months, 9.1 months, and 3.1 months, respectively. In a phase II trial, the antitumor efficacy of Lapatinib was evaluated in 33 SACC patients [144]. Among the 19 assessable patients with DP, no objective responses were recorded. However, it is noteworthy that 79% of patients exhibited SD, with 47% maintaining SD for six months or longer. In contrast, 21% of patients experienced DP.

These therapeutic agents, while effective in stabilizing tumors, largely fail to prevent tumor progression. The development of novel pharmaceutical agents and innovative approaches is necessary to elevate the standard of SACC treatment beyond mere disease stabilization.

## 12. Future Directions and Conclusions

SACC is the predominant malignant tumor affecting minor salivary glands, yet it is a relatively rare condition worldwide, with an annual occurrence of merely 3–4.5 cases per million individuals [4]. Despite the fact that this type of tumor is not commonly encountered, its biological behavior is unpredictable and slow growing. Nevertheless, it has a marked inclination to grow along nerve fibers and invade lymphatic vessels, rendering it a highly invasive and aggressive tumor. As a result, patients with these tumors generally have a bleak outlook, which has not shown much improvement in recent years. This is largely due to the limited availability of successful chemotherapeutic regimens that can prevent invasion and metastasis [147]. The metastasis of SACC appears to be governed by the expression and alteration of the extracellular matrix. This, in turn, is accountable for promoting the EMT process in tumor cells [148].

Cancer cells can undergo partial EMT, which means that they can have the characteristics of both mesenchymal and epithelial cells [149]. Instead, metastatic cancer cells with mesenchymal features must undergo MET, which is the reverse transition from mesenchymal to epithelial, at the secondary site to survive and mimic the primary tumors in various cancer types. Previous studies have presented the advantages of MET in breast cancer, small-cell lung cancer, and ovarian cancer, but not many studies have investigated its role in SACC metastases [150,151,152]. Hence, it is essential to have a more comprehensive understanding of the molecular mechanisms that control cell plasticity induced by EMT or MET, and how they contribute functionally to the progression of malignant tumors. However, the main challenge appears to be accurately measuring the degree of “partial EMT” in different disease conditions and accurately capturing these constantly changing states. Despite the intricate nature of the mechanisms involved in EMT, certain aspects and connections have become more apparent over time.

Through several inquiries dedicated to EMT research in SACC, promising targetable biomarkers have been identified that hold potential for future assessment of the tumor’s invasiveness and propensity for metastasis. According to the aforementioned findings, the induction of the TGF-beta signaling pathway, activation of the MYB oncogene, a hypoxic microenvironment, alteration in the expression of miRs (e.g., miR-9, miR-138, miR-155, and miR-222), and increased expression of c-Kit, appear to be important factors that might advance the progression of EMT in SACC. These conflicting results regarding established mesenchymal and epithelial markers, as well as novel EMT mediators in SACC, highlight the fact that the precise molecular mechanisms underlying the interplay between different EMT pathways in this disease are yet to be fully understood.

To sum up, it is crucial to identify the different cellular states that are linked to EMT plasticity in order to pinpoint EMT components that can be targeted and design treatment plans that can efficiently eliminate cells undergoing EMT, as this is the root cause of treatment resistance. Our review delving into the intricate mechanisms involved in the progression from primary tumor cell dissociation through EMT to the development of metastasis might provide encouraging prospects for the future.

## Figures and Tables

**Table 1:** Expression of different EMT-TFs, their original function, and participation in epithelial–mesenchymal transition (EMT) in salivary adenoid cystic carcinoma (SACC). DM: distant metastasis; PNI: perineural invasion; TNM: Tumor, Node, Metastasis.

EMT-TF	Original Function	Role of EMT in SACC	References
TWIST1/TWIST2	- Member of the basic helix–loop–helix transcription factor family; - Participates in cell lineage determination and differentiation during embryonic development; - Expression decreases following birth; - Expression is found in precursor cells of adult tissues.	- Strongly expressed in SACC, - Associated with PNI, local regional recurrence, DM, and SACC with a solid pattern, - Reduction in TWIST levels results in reduced cancer cell migration, invasion, and PNI ability	[13,36,37,38,39,40,41,42,43,44,45]
SNAIL	- First discovered and plays a pivotal role in Drosophila melanogaster; - Involved in cell survival, immune regulation, and stem cell biology; - Three members of Snail zinc-finger transcription factors have been identified in vertebrates: Snail1 (Snail), Snail2 (Slug), and Snail3 (Smuc).	- Heterogeneous reactivity in SACC; - More distinct nuclear pattern in solid variant and invasive tumor front; - Associated with PNI, local and regional recurrence, DM, and unfavorable prognosis.	[37,46,47,48,49,50,51]
SLUG	- Member of Snail zinc-finger transcription factors; - First described in neural crest and mesoderm in chick embryos; - Can be found in the majority of adult tissues, which may assist in preserving their proper function following birth.	- Strongly expressed in SACC; - Related to advanced TNM stages, higher risk of PNI and DM, and poorer prognosis.	[37,52,53,54]
ZEB1/ZEB2	- Contributes to regular embryonic development, primarily by triggering EMT.	- Highly expressed in SACC; - Linked to higher risk of PNI, DM, and poorer prognosis.	[37,55,56,57]

**Table 2:** Completed and ongoing phase I/II clinical trials investigating therapies influencing the epithelial–mesenchymal transition (EMT) axis in salivary adenoid cystic carcinoma (SACC). VEGFR: vascular endothelial growth factor receptor; FGFR: fibroblast growth factor receptor; PDGFR: platelet-derived growth factor receptor; EGFR: epidermal growth factor receptor; HER2: human epidermal growth factor receptor 2; GSK3β: glycogen synthase kinase-3 beta; mTOR: mammalian target of rapamycin; HIF: hypoxia-inducible factor.

Intervention	Targets	EMT-Axis	Phase	Status	References
Lenvatinib	VEGFR, FGFR, PDGFR, RET, KIT	Pi3K/AKT/GSK3β/Snail	II	Completed	NCT02860936
Dovitinib	VEGFR, FGFR, PDGFR, RET, KIT	Pi3K/AKT/GSK3β/Snail	II	Completed	NCT01678105
Sunitinib	VEGFR, PDGFR, KIT	Pi3K/AKT/GSK3β/Snail	II	Completed	NCT00886132
Amivantamab	EGFR, MET	Pi3K/AKT/GSK3β/Snail, Src/ERK/Slug	II	Recruiting	NCT05074940
Dasatinib	BCR-ABL, SRC	Pi3K/AKT/GSK3β/Snail, Src/ERK/Slug	II	Completed	NCT00859937
Lapatinib	HER2, EGFR	Pi3K/AKT/GSK3β/Snail, Src/ERK/Slug	II	Completed	NCT00095563
9-ING-41 Plus Carboplatin	GSK3β	WNT/β-catenin/Snail	II	Recruiting	NCT05010629
Akt Inhibitor MK2206	Akt	Pi3K/AKT/GSK3β/Snail	II	Completed	NCT01604772
Imatinib	BCR-ABL, KIT	Pi3K/AKT/GSK3β/Snail	II	Completed	NCT00045669
Trastuzumab	HER2	Pi3K/AKT/GSK3β/Snail, Src/ERK/Slug	II	Completed	NCT00004163
Gefitinib	EGFR	Pi3K/AKT/GSK3β/Snail, Src/ERK/Slug	I	Completed	NCT00068497
Erlotinib and Cetuximab with or without Bevacizumab	EGFR, VEGFR	Pi3K/AKT/GSK3β/Snail, Src/ERK/Slug	I	Completed	NCT00101348
Cetuximab and Everolimus	EGFR, mTOR, HIF	Pi3K/AKT/GSK3β/Snail, Src/ERK/Slug, HIF-1α	I	Completed	NCT01637194
